# Risk of loco-regional recurrence and distant metastases of patients with invasive breast cancer up to ten years after diagnosis – results from a registry-based study from Germany

**DOI:** 10.1186/s12885-019-5710-5

**Published:** 2019-05-30

**Authors:** Bernd Holleczek, Christa Stegmaier, Julia C. Radosa, Erich-Franz Solomayer, Hermann Brenner

**Affiliations:** 1 Division of Clinical Epidemiology and Aging Research, German Cancer Research Center (DKFZ), INF 581, 69120 Heidelberg, Germany; 2 Saarland Cancer Registry, Präsident Baltz-Straße 5, 66119 Saarbrücken, Germany; 3 Department of Gynecology and Obstetrics, Saarland University Hospital, Kirrberger Straße 100, 66421 Homburg/Saar, Germany; 4 Division of Preventive Oncology, German Cancer Research Center (DKFZ) and National Center for Tumor Diseases (NCT), INF 460, 69120 Heidelberg, Germany; 5 German Cancer Consortium (DKTK), German Cancer Research Center (DKFZ), INF 280, 69120 Heidelberg, Germany

**Keywords:** Invasive breast cancer, Loco-regional recurrence, Distant metastases, Cumulative incidence, Cancer registry, Population-based, Germany

## Abstract

**Background:**

Population-based estimates of the long-term risk of loco-regional recurrence and distant metastases of breast cancer (BRC) patients are scant, as most published studies used hospital-based cohorts or participants of clinical trials. This work aims to extend available knowledge by providing population-based long-term estimates of the cumulative risk of BRC recurrence up to 10 years after diagnosis.

**Methods:**

Data from the population-based Saarland Cancer Registry were used and included 9359 female patients with primary invasive BRC diagnosed between 1999 and 2009. Estimates of the cumulative incidence (CI) of BRC recurrence were derived for patients who had received local surgery with free resection margins by type of recurrence and stratified by age, tumor characteristics and major treatment options, taking into account mortality from any cause as a competing risk.

**Results:**

The 10-year CI of BRC recurrence was 16%. For loco-regional recurrence and distant metastases alone it was 8 and 11%, respectively. The estimates showed substantial variation and were particularly increased if tumors were advanced (T1/2N+ 23%, T3/4N0 24%, T3/4N+ 34%), of high grade (23%), or of ‘HER2/neu positive’ (28%) or ‘triple negative’ subtype (23%), respectively.

**Conclusions:**

The derived estimates reflect the risk of ‘real world’ patients and may therefore extend available knowledge. These data are thus of great relevance for clinicians, their patients and researchers. The study likewise demonstrated the usefulness of cancer registries for a population-based monitoring of the effectiveness of cancer care in terms of disease recurrence as a major treatment related outcome measure.

**Electronic supplementary material:**

The online version of this article (10.1186/s12885-019-5710-5) contains supplementary material, which is available to authorized users.

## Background

Breast cancer (BRC) is the most common invasive cancer in women worldwide [1] and the overall prognosis of BRC patients in industrialized countries is favorable [2, 3]. However, significant proportions of patients suffer from recurrence, e.g. a trial from the US cited in clinical practice guidelines (CPG) reported occurrence of loco-regional recurrence and distant metastases over 10 years in one out of every six patients and in one out of every three patients who had been diagnosed between 1975 and 1994, respectively [4]. BRC recurrence may occur in the ipsilateral breast or chest wall after surgery, regional lymph nodes (including ipsilateral axillary, infraclavicular, internal mammary, or supraclavicular nodes), as well as distant sites and organs.

Factors associated with increased risk of loco-regional recurrence include young age at diagnosis, advanced tumor size, involvement of regional lymph nodes, high grade, vascular invasion, and omitting an indicated adjuvant radiotherapy [5]. Surrogate definitions based on immunohistochemical measurements of the expression of hormone receptors (HR), human epidermal growth factor receptor 2 (HER2/neu), and other markers such as the Ki-67 antigen are used to classify BRC and correlate well with genetically different BRC subtypes [6, 7], which are associated with the risk of recurrence and outcome in addition to classic prognostic factors [8, 9].

Treatment options of potentially curable loco-regional recurrence include complete surgical resection of the recurrent tumor, radiotherapy and systemic treatment based on histological examination of the cancerous tissue and re-staging. Occurring distant metastases are generally treated with palliative intent and therapy includes systemic treatment, radiotherapy or resection of metastases. Any treatment of recurrent BRC should be based on an interdisciplinary approach [10–12].

Data on the long-term risk of BRC recurrence which were derived from population-based samples of patients are scant, as the vast majority of published studies used selected patient samples such as hospital-based cohorts (e.g. [7, 13–15]) or participants of clinical trials (e.g. [4, 16, 17]). Furthermore, most studies were restricted to 5 years of follow-up only. This study from Germany aims to overcome these shortcomings by providing long-term estimates of the cumulative risk of loco-regional recurrence and distant metastases up to 10 years after diagnosis according to age, tumor characteristics and major treatment options derived from a population-based sample of BRC patients who had received surgery with free resection margins.

## Methods

Data from the population-based Saarland Cancer Registry (CR) were used and included records of 9359 female patients with primary invasive BRC (ICD-10 code: C50) diagnosed between 1999 and 2009. Saarland is a federal state in Southwest Germany with a population of approximately 1.02 million people in 2009. The CR is in operation since 1968 and its case ascertainment is regularly estimated to be almost complete (e.g. [18, 19]). In Saarland, cancer reporting is mandatory by law. The CR obtains cancer notifications from hospitals, radiotherapy departments, outpatient clinics, screening programs, and doctors in private practice as well as reports from pathology laboratories. In addition to notifications of newly diagnosed cancers, the CR obtains notifications of recurrent cancer from the above listed sources on the occasion of their diagnosis and treatment. The vital status of the patients is regularly ascertained using death certificates from local health authorities and records from population registries. The Saarland CR applies the rules of recording multiple primaries as recommended by the International Association of Cancer Registries [20]. Loco-regional recurrences and distant metastases of a tumor are recorded, if stated as such in a case summary or a pathology report. Accordingly, tumors of the contralateral breast and multiple primary tumors of the breast were not considered as cancer recurrences.

The following classifications of socio-demographic and tumor characteristics as well as administered treatments were used in the analyses: age at diagnosis (< 70, ≥ 70 years), clinical stage (T1/2N0, T1/2N+, T3/4N0, T3/4N+, M1), morphologic type of tumor (invasive ductal, invasive lobular, mixed type, other), histopathologic grade (G1, G2, G3/4), HR expression (positive (including mixed: either estrogen or progesterone receptor positive), negative), HER2/neu expression (positive (including borderline results with further examinations), negative), definitive local surgery (breast conserving surgery (BCS), mastectomy, none), provision of radiotherapy (yes, none), chemotherapy (yes, none), hormonal (anti-estrogen) therapy (yes, none) and targeted treatment (TT) with monoclonal antibodies (yes, none), residual tumor after surgery (no residual tumor (R0), microscopic or macroscopic residual tumor (R1/2)), and source of registration (notification at lifetime, death certificate only). Further details on the data collection may be found elsewhere [21]. Information on the HR status and HER2/neu expression was extracted from available pathology reports and case summaries. HR status was based on immunohistochemistry. According to CPGs, expression of estrogen and progesterone receptors was considered, if more than 10% of cancerous cells showed immunohistochemical nuclear staining (for two tumors, biochemistry was used for quantitative measurements) [22, 23]. HER2/neu positivity was based on immunohistochemical tests with score 3+ or in 28 cases with unclear results after fluorescence or chromogenic in situ hybridization [22, 23]. Based on the available measurements of HR expression and HER2/neu expression, tumors were classified as ‘HR positive’, ‘HER2/neu positive’ (HER2/neu positive, but HR negative) and ‘triple negative’ (both HR and HER2/neu negative) [12, 24]. It has been shown, that these immunohistochemical definitions largely describe the major intrinsic BRC subtypes ‘luminal like’, ‘HER2/neu like’ and ‘basal like’, respectively [6, 7].

During the period of diagnosis, the national CPG recommended BCS followed by radiotherapy or mastectomy for early stage BRC or mastectomy followed by radiotherapy for locally advanced tumors (guidelines recommended surgery with clear resection margins), sentinel node biopsy or dissection of regional lymph nodes for proper staging, adjuvant chemotherapy for patients with involved lymph nodes or HR negative tumors, hormonal treatment for patients with HR positive tumors and TT with monoclonal antibodies for patients with HER2/neu expressed tumors. In case of advanced tumors, systemic treatment was recommended prior to surgery and radiotherapy. For elderly patients, a treatment comparable to younger patients was recommended taking altered organ functions and co-morbidity into account [25].

Descriptive analyses were used to summarize the patients with regard to age and tumor characteristics. The study sample included all BRC patients without distant metastases at the time of diagnosis. A summary of the provided local and systemic treatments was derived. For the analysis of the risk of recurrence, information on its type (loco-regional recurrence, distant metastases), date of first occurrence and date of death was used.

Survival observations were right censored, if no event was observed until end of follow-up (31 December 2014, at the latest) and survival times between diagnosis and first occurrence of local recurrence, distant metastases, death, or end of follow-up were derived. The analyses of the risk of BRC recurrence included patients who received local surgery with free resection margins (R0) without any BRC recurrence within 3 months and for whom follow-up was available (*n* = 5311, 62% of the study sample). This 3 month cutoff period was chosen to allow for any changes in the staging during diagnostic work-up or initial treatment to avoid misclassification of such changes as recurrences. Five- and 10-year estimates of the cumulative incidence (CI) were derived by type of recurrence and stratified by age, tumor characteristics and major treatment options, taking into account mortality from any cause as a competing risk. The used estimator of the CI is based on a generalization of the Kaplan-Meier estimator and quantifies the probability that the event under study will occur before any specified time in the presence of competing risks [26, 27]. Along with point estimates, 95% confidence intervals of the CI were derived [28]. The R Language and Environment for Statistical Computing (release 3.1.3) [29] and the cmprsk extension package [30] were used for data preparation, statistical analyses and visualization. *P*-values of a two sided test of the equality of CI curves across subsamples [30] were derived and considered as a statistically significant result, if < 0.05.

## Results

Table 1 presents the distribution of tumor characteristics of the patients. Overall, 56% had a T1/2N0 tumor, 69% had an invasive ductal carcinoma, and 64% had a tumor of intermediate grade. Of the tumors, 84 and 25% showed expression of HR and HER2/neu, respectively. Mean age at diagnosis was 62.7 years, 2.4% of the patients presented with bilateral BRC and mean observation time was 10.3 years. Among elderly patients, higher proportions of tumors with advanced stage, HR expression and without HER2/neu expression were observed.

**Table 1:** Tumor characteristics of female patients with primary invasive breast cancer overall and by age

Category		Overall		< 70 years		≥ 70 years	
		N	% ^a^	N	% ^a^	N	% ^a^
Overall		9359		6138	65.6	3221	34.4
Clinical stage	M1	658	7.0	377	6.1	281	8.7
Death certificate only notification		183	2.0	36	0.6	147	4.6
Study sample		8518	91.0	5725	93.3	2793	86.7
Laterality	available	8508	99.9	5716	99.8	2792	100.0
	unilateral	8306	*97.6*	5600	*98.0*	2706	*96.9*
	synchronous bilateral^b^	202	*2.4*	116	*2.0*	86	*3.1*
Clinical stage	available	7447	87.4	5312	92.8	2135	76.4
	T1/2N0	4132	*55.5*	3011	*56.7*	1121	*52.5*
	T1/2N+	2379	*31.9*	1787	*33.6*	592	*27.7*
	T3/4N0	205	*2.8*	111	*2.1*	94	*4.4*
	T3/4N+	731	*9.8*	403	*7.6*	328	*15.4*
Microscopically verified		8482	99.6	5723	100.0	2759	98.8
Morphology	available	8477	99.5	5720	99.9	2757	98.7
	invasive ductal	5883	*69.4*	4053	*70.9*	1830	*66.4*
	invasive lobular	1190	*14.0*	773	*13.5*	417	*15.1*
	mixed type	654	*7.7*	465	*8.1*	189	*6.9*
	other	750	*8.8*	429	*7.5*	321	*11.6*
Histopathologic grade	available	8216	96.5	5564	97.2	2652	95.0
	1	610	*7.4*	405	*7.3*	205	*7.7*
	2	5232	*63.7*	3458	*62.1*	1774	*66.9*
	3/4	2374	*28.9*	1701	*30.6*	673	*25.4*
HR expression	available	7424	87.2	5048	88.2	2376	85.1
	positive^c^	6235	*84.0*	4152	*82.3*	2083	*87.7*
	negative	1189	*16.0*	896	*17.7*	293	*12.3*
HER2/neu expression	available	5938	69.7	4051	70.8	1887	67.6
	positive^d^	1466	*24.7*	1054	*26.0*	412	*21.8*
	negative	4472	*75.3*	2997	*74.0*	1475	*78.2*
Subtype based on immunohistochemical surrogates	HR and HER2/neu status available	7197	84.5	4883	85.3	2314	82.8
	‚HR positive‘	6235	*86.6*	4152	*85.0*	2083	*90.0*
	‚HER2/neu positive‘^e^	368	*5.1*	278	*5.7*	90	*3.9*
	‚triple negative’^f^	594	*8.3*	453	*9.3*	141	*6.1*
Follow-up available		8380	98.4	5634	98.4	2746	98.3
		years		years		years	
Mean observation time		10.3		10.4		10.3	

The overall study sample includes all breast cancer patients (ICD-10 code: C50) from Saarland diagnosed between 1999 and 2009. *T, N* = T and N category of TNM classification, *HR* = hormone receptor, *HER2/neu* = human epidermal growth factor receptor 2. ^a^figures printed in normal text represent numbers and proportions of the overall cohort or study sample, figures printed in italic text represent proportions of cases of the respective sample with available information, ^b^tumors were classified as synchronous bilateral if the time interval between their detection was ≤3 months, ^c^includes tumors with mixed HR expression (either estrogen or progesterone receptor positive), ^d^includes tumors with borderline expression of HER2/neu, ^e^HER2/neu positive, but HR negative, ^f^both HR negative and HER2/neu negative

Table 2 shows the provision of local and systemic treatments. Overall, 64 and 35% of the patients received BCS and mastectomy, respectively. Among patients with T1/2N0 and T1/2N+ tumors, 71 and 73% received both surgery and radiotherapy. Of the patients with T3/4 tumors, 46% received mastectomy followed by radiotherapy. After local surgery, 95% of the patients had free resection margins (R0). Of the patients with nodal positive or HR negative tumors, 70% received chemotherapy and 85% of the patients with HR positive tumors received hormonal treatment. Of the patients with HER2/neu positive tumors, 25% received TT. The provision of treatments varied markedly by age. Elderly patients received mastectomy more often and recommended radiotherapy, chemotherapy and TT less often.

**Table 2:** Provision of local and systemic treatments to breast cancer patients

Category/treatment	Sample	Overall		< 70 years		≥ 70 years	
		N	% ^a^	N	% ^a^	N	% ^a^
Overall	study sample (8518)	8518		5725		2793	
Information on local treatment available	study sample (8518)	6634	77.9	4548	79.4	2086	74.7
BCS	patients with information on surgery (6634)	4245	*64.0*	3225	*70.9*	1020	*48.9*
Mastectomy		2290	*34.5*	1300	*28.6*	990	*47.5*
None		99	*1.5*	23	*0.5*	76	*3.6*
BCS	patients with T1/2N0 tumors and information on surgery (3435)	244	*7.1*	134	*5.4*	110	*11.7*
BCS + RT		2427	*70.7*	1925	*77.3*	502	*53.2*
Mastectomy		754	*22.0*	427	*17.1*	327	*34.6*
BCS or mastectomy + RT	patients with T1/2N+ tumors and information on surgery (1995)	1452	*72.8*	1162	*78.2*	290	*57.0*
Mastectomy + RT	patients with T3/4 tumors and information on surgery (755)	350	*46.4*	232	*55.6*	118	*34.9*
Residual status available	patients with surgery (6535)	5709	87.4	3972	87.8	1737	86.4
R0		5409	*94.7*	3773	*95.0*	1636	*94.2*
R1/2		300	*5.3*	199	*5.0*	101	*5.8*
CT information available	study sample (8518)	6941	81.5	4961	86.7	1980	70.9
CT	patients with N+ or HR negative tumors and information on CT usage (3472)	2429	*70.0*	2171	*84.9*	258	*28.2*
HT information available	study sample (8518)	6740	79.1	4709	82.3	2031	72.7
HT	patients with HR positive tumors ^b^ and information on HT usage (5122)	4356	*85.0*	3031	*85.4*	1325	*84.3*
TT information available	study sample (8518)	4786	56.2	3375	59.0	1411	50.5
TT	patients with HER2/neu positive tumors ^c^ and information on TT usage (839)	208	*24.8*	183	*29.3*	25	*11.7*

The study sample includes female patients with primary invasive breast cancer (ICD-10 code: C50) without distant metastases from Saarland diagnosed between 1999 and 2009. *BCS* breast conserving surgery, *RT* radiotherapy, *T, N* T and N category of TNM classification, *CT* chemotherapy, *HR* hormone receptor, *HT* hormonal (anti-estrogen) treatment, *TT* targeted treatment (trastuzumab), *HER2/neu* human epidermal growth factor receptor 2. ^a^figures printed in normal text represent numbers and proportions of the study sample, figures printed in italic text represent proportions of cases of the respective sample with available information, ^b^includes tumors with mixed HR expression (either estrogen or progesterone receptor positive), ^c^includes tumors with borderline expression of HER2/neu

Table 3 presents the 5- and 10-year CI of cancer recurrence (both loco-regional recurrence and distant metastases) of BRC patients with local R0 resection by age, tumor characteristics and provided recommended treatments. Overall 5- and 10-year CI of BRC recurrence was 10 and 16%, respectively (unless otherwise stated, the 10-year estimate of the CI will be reported subsequently). The CI was higher of patients aged < 70 years (17% vs. 13%; *p* < 0.001). It was 9% in patients with T1/2N0 tumors, 23 and 24% in patients with T1/2N+ and T3/4N0 tumors and 34% in patients with T3/4N+ tumors, respectively (*p* < 0.001). It was 6, 14 and 23% in patients with low, intermediate and high grade carcinomas (*p* < 0.001). Little variation of the CI was observed by tumor morphology. The CI of cancer recurrence of patients with tumors of the subtype ‘HR positive’ was approximately half compared to the CI of patients with subtype ‘HER2/neu positive’ and ‘triple negative’ tumors (14% vs. 28 and 26%, *p* < 0.001), respectively. The overall 10-year risk of BRC recurrence of patients with and without HR expression was 27 and 14%, respectively (*p* < 0.001, the risk did not vary significantly between patients with tumors showing expression of estrogen or progesterone receptors, data not shown). The overall 10-year risk of BRC recurrence of patients with a HER2/neu positive tumor was 22%, varying between 28% among those with HR negative tumor and 20% among those with a HR positive tumor (*p* < 0.001, data not shown).

**Table 3:** Five- and 10-year cumulative incidence of cancer recurrence of breast cancer patients with local R0 resection by age, tumor characteristics and provided recommended treatments

Category/treatment		N	5-year CI		10-year CI		*p*-value
			PE	95% CI	PE	95% CI	
Overall		5311	10.4	[9.6, 11.3]	15.9	[14.8, 17.0]	
Age	< 70 years	3714	11.2	[10.2, 12.3]	17.2	[16.0, 18.6]	< 0.001
	≥ 70 years	1597	8.7	[7.4, 10.2]	12.6	[11.0, 14.5]	
Clinical stage	T1/2N0	2918	5.6	[4.8, 6.5]	9.4	[8.3, 10.7]	< 0.001
	T1/2N+	1613	14.8	[13.1, 16.6]	23.1	[20.9, 25.5]	
	T3/4N0	124	16.1	[10.8, 24.2]	23.5	[16.7, 33.1]	
	T3/4N+	409	26.9	[22.9, 31.6]	34.3	[29.8, 39.6]	
	missing	247	9.0	[6.0, 13.4]	9.9	[6.7, 14.5]	
Morphology	invasive ductal	3690	10.9	[10.0, 12.0]	16.2	[14.9, 17.5]	0.075
	invasive lobular	758	9.7	[7.8, 12.0]	17.8	[15.0, 21.1]	
	mixed type	459	9.0	[6.7, 12.0]	14.3	[11.3, 18.2]	
	other	404	9.0	[6.6, 12.3]	11.1	[8.3, 14.9]	
Histopathologic grade	1	447	2.7	[1.5, 4.7]	5.9	[3.9, 8.9]	< 0.001
	2	3332	8.5	[7.6, 9.5]	13.9	[12.6, 15.2]	
	3/4	1497	16.8	[15.0, 18.8]	23.0	[20.9, 25.4]	
	missing	35	17.6	[8.4, 36.8]	21.8	[11.0, 43.1]	
HR expression	positive^a^	4132	8.3	[7.5, 9.2]	14.0	[12.9, 15.2]	< 0.001
	negative	775	22.8	[20.0, 26.0]	27.1	[24.0, 30.5]	
	missing	404	9.0	[6.6, 12.3]	12.6	[9.4, 16.8]	
HER2/neu expression	positive^b^	972	13.3	[11.3, 15.6]	21.7	[18.9, 24.8]	< 0.001
	negative	2941	9.6	[8.6, 10.7]	14.0	[12.7, 15.5]	
	missing	1398	10.2	[8.7, 11.9]	15.6	[13.8, 17.7]	
Subtype based on immunohistochemical surrogates	‚HR positive‘	4132	8.3	[7.5, 9.2]	14.0	[12.9, 15.2]	< 0.001
	‚HER2/neu positive‘^c^	234	22.8	[18.0, 28.9]	28.0	[22.4, 34.9]	
	‚triple negative‘^d^	376	23.2	[19.3, 27.9]	25.6	[21.4, 30.5]	
	missing	569	12.7	[10.3, 15.8]	17.8	[14.7, 21.6]	
BCS	T1/2N0	198	5.6	[3.1, 10.0]	7.7	[4.4, 13.4]	
BCS + RT	T1/2N0	2114	4.6	[3.8, 5.6]	8.4	[7.2, 9.8]	
Mastectomy	T1/2N0	606	9.1	[7.1, 11.8]	13.5	[10.9, 16.7]	
(BCS + RT or mastectomy) + HT	T1/2N0 HR positive^a^	1749	3.9	[3.1, 4.9]	8.2	[6.9, 9.8]	
(BCS + RT or mastectomy) + CT	T1/2N0 HR negative	277	12.3	[9.0, 16.8]	15.5	[11.6, 20.6]	
(BCS + RT or mastectomy) + HT (if HR positive) + CT (if HR negative) + TT	T1/2N0 HER2/neu positive	73	2.7	[0.7, 10.9]	2.7	[0.7, 10.9]	
(BCS or mastectomy) + RT + CT + HT	T1/2N+ HR positive^a^	554	12.0	[9.6, 15.1]	19.5	[16.1, 23.7]	
(BCS or mastectomy) + RT + CT	T1/2N+ HR negative	156	28.4	[22.1, 36.5]	36.9	[29.6, 46.0]	
(BCS or mastectomy) + RT + CT + HT (if HR positive) + TT	T1/2N+ HER2/neu positive	51	19.6	[11.2, 34.4]	21.9	[12.9, 37.2]	
MAST + RT + CT + HT	T3/4 HR positive^a^	88	26.1	[18.4, 37.2]	34.0	[24.7, 46.9]	
MAST + RT + CT	T3/4 HR negative	50	48.0	[35.8, 64.3]	52.2	[39.8, 68.5]	

The sample includes female patients with primary invasive breast cancer (ICD-10 code: C50) without distant metastases from Saarland diagnosed between 1999 and 2009. *CI* cumulative incidence, *PE* point estimate of CI, *95% CI* 95% confidence interval of PE, *T, N* T and N category of TNM classification, *HR* hormone receptor, *HER2/neu* human epidermal growth factor receptor 2, *BCS* breast conserving surgery, *RT* radiotherapy, *CT* chemotherapy, *HT* hormonal (anti-estrogen) treatment, *TT* targeted therapy (trastuzumab). ^a^includes tumors with mixed HR expression (either estrogen or progesterone receptor positive), ^b^includes tumors with borderline expression of HER2/neu, ^c^HER2/neu positive, but HR negative, ^d^both HR negative and HER2/neu negative

The 10-year CI of BRC recurrence of patients with T1/2N0 tumors was 8% if they had received BCS, but 14% if they had received a mastectomy. Patients with T1/2N0 tumors who had undergone BCS followed by radiation therapy (RT) or mastectomy had a CI of 8% if HR positive tumors were treated with hormonal therapy and of 16% if HR negative tumors were treated with chemotherapy, respectively. Patients with T1/2N+ tumors who had received BCS or mastectomy followed by RT had a CI of 20% if having a HR positive tumor and treated with both chemotherapy and hormonal therapy and of 37% if having a HR negative tumor and treated with chemotherapy alone, respectively. Patients with locally advanced T3/4 tumors who had received mastectomy, radiotherapy and chemotherapy had a CI of 52% if the tumors were HR negative, but if the tumors were HR positive and the patients additionally had received hormonal therapy, the CI was 34%.

Patients with HER2/neu positive T1/2N0 tumors had a 10-year risk of tumor recurrence of 3% if they received trastuzumab (*N* = 73) compared to 11% if they did not receive trastuzumab (*N* = 159) in addition to other recommended local and systemic treatments (*p* = 0.074). The overall 10-year risk of BRC recurrence of patients with T1/2N+ tumors showing HER2/neu expression was 22% if they received (*N* = 51) and 32% if they did not receive (*N* = 73) TT (*p* = 0.862, data not shown). The patients of the entire study sample and those included in the analysis of the risk of BRC recurrence strongly resembled both in terms of age and tumor characteristics (Table 4).

**Table 4:** Distribution of age and tumor characteristics of breast cancer patients of the study sample (*N* = 8511) and those included in the analysis of the risk of BRC recurrence (*N* = 5311)

Category		Study sample		Patients with a local R0 resection, available follow-up and without recurrence within 3 months	
		N	% ^a^	N	% ^a^
Overall		8518		5311	
Age	< 70 years	5725	67.2	3714	69.9
	≥ 70 years	2793	32.8	1597	30.1
Laterality	available	8508	99.9	5307	99.9
	unilateral	8306	*97.6*	5198	*97.9*
	synchronous bilateral ^b^	202	*2.4*	109	*2.1*
Clinical stage	available	7447	87.4	5064	95.3
	T1/2N0	4132	*55.5*	2918	*57.6*
	T1/2N+	2379	*31.9*	1613	*31.9*
	T3/4N0	205	*2.8*	124	*2.4*
	T3/4N+	731	*9.8*	409	*8.1*
Microscopically verified		8482	99.6	5311	100.0
Morphology	available	8477	99.5	5311	100.0
	invasive ductal	5883	*69.4*	3690	*69.5*
	invasive lobular	1190	*14.0*	758	*14.3*
	mixed type	654	*7.7*	459	*8.6*
	other	750	*8.8*	404	*7.6*
Histopathologic grade	available	8216	96.5	5376	99.3
	1	610	*7.4*	447	*8.5*
	2	5232	*63.7*	3332	*63.2*
	3/4	2374	*28.9*	1497	*28.4*
HR expression	available	7424	87.2	4907	92.4
	positive ^c^	6235	*84.0*	4132	*84.2*
	negative	1189	*16.0*	775	*15.8*
HER2/neu expression	available	5938	69.7	3913	73.7
	positive ^d^	1466	*24.7*	972	*24.8*
	negative	4472	*75.3*	2941	*75.2*
Subtype based on immunohistochemical surrogates	HR and HER2/neu status available	7197	84.5	4742	89.3
	’HR positive‘	6235	*86.6*	4132	*87.1*
	‚HER2/neu positive‘^e^	368	*5.1*	234	*4.9*
	‚triple negative‘^f^	594	*8.3*	376	*7.9*
Follow-up available		8380	98.4	5311	100.0
		years		years	
Mean observation time		10.3		10.2	

The sample includes female patients with primary invasive breast cancer (*BRC*) (ICD-10 code: C50) without distant metastases from Saarland diagnosed between 1999 and 2009. *T, N* T and N category of TNM classification, *HR* hormone receptor, *HER2/neu* human epidermal growth factor receptor 2. ^a^ figures printed in normal text represent numbers and proportions of the study sample and of patients with a local R0 resection who were included in the analysis of the risk of BRC recurrence, figures printed in italic text represent proportions of patients of the respective sample with available information, ^b^ tumors were classified as synchronous bilateral if the time interval between their detection was ≤3 months, ^c^ includes tumors with mixed HR expression (either estrogen or progesterone receptor positive), ^d^ includes tumors with borderline expression of HER2/neu, ^e^ HER2/neu positive, but HR negative, ^f^ both HR negative and HER2/neu negative

**Fig. 1:**
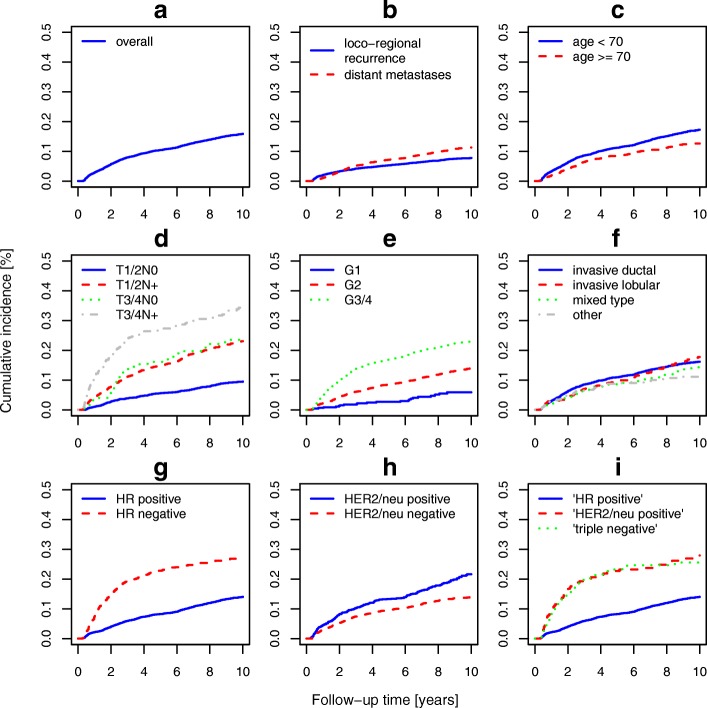
Ten-year cumulative incidence curves of cancer recurrence of female patients with primary invasive breast cancer. Cumulative incidence of cancer recurrence of breast cancer patients (ICD-10 code: C50) without distant metastases from Saarland after local R0 resection and diagnosed between 1999 and 2009 overall (subfigure **a**), by type of recurrence (**b**), age (**c**), clinical stage (**d**), histopathologic grade (**e**), morphologic type (**f**), hormone receptor expression (**g**), HER2/neu expression (**h**), and subtype based on immunohistochemical surrogates (**i**). *T, N* T and N category of TNM classification, *HR* hormone receptor, *HER2/neu* human epidermal growth factor receptor 2

Tables 5 and 6 present estimates of the 5- and 10-year CI by type of recurrence. The overall 10-year CI of loco-regional recurrence and distant metastases after surgery with free resection margins was 8 and 11%, respectively. The CI of loco-regional recurrence and distant metastases of patients with small and localized tumors was of similar size. However, if regional lymph nodes were involved, the risk of distant metastases increased disproportionally, i.e. the 10-year CI of loco-regional recurrence and distant metastases of patients with T1/2 tumors with positive lymph nodes was 10 and 18%, respectively. The overall 10-year risk of metastasis to distant sites of patients who did not have clear resection margins after definitive surgery was 27% and thus 2.4-fold increased compared to those with free resection margins (*p* < 0.001, data not shown). Figure 1 depicts curves of 10-year CI of BRC recurrence by type of recurrence, age, and tumor characteristics.

**Table 5:** Five- and 10-year cumulative incidence of loco-regional recurrence of breast cancer patients with local R0 resection by age, tumor characteristics and provided recommended treatments

Category/treatment		N	5-year CI		10-year CI		*p*-value
			PE	95% CI	PE	95% CI	
Overall		5311	5.4	[4.8, 6.0]	7.8	[7.0, 8.6]	
Age	< 70 years	3714	5.7	[5.0, 6.5]	8.5	[7.6, 9.5]	0.007
	≥ 70 years	1597	4.5	[3.6, 5.6]	6.1	[4.9, 7.4]	
Clinical stage	T1/2N0	2918	2.8	[2.3, 3.5]	5.1	[4.3, 6.1]	< 0.001
	T1/2N+	1613	7.3	[6.1, 8.7]	10.3	[8.8, 12.0]	
	T3/4N0	124	8.9	[5.0, 15.7]	13.0	[8.0, 21.1]	
	T3/4N+	409	13.9	[10.9, 17.7]	15.1	[12.0, 19.0]	
	missing	247	6.9	[4.4, 11.0]	7.8	[5.1, 12.1]	
Morphology	invasive ductal	3690	5.7	[5.0, 6.5]	8.1	[7.2, 9.1]	0.395
	invasive lobular	758	4.5	[3.2, 6.3]	8.0	[6.2, 10.5]	
	mixed type	459	4.1	[2.7, 6.4]	6.0	[4.1, 8.7]	
	other	404	5.5	[3.7, 8.3]	6.5	[4.4, 9.5]	
Histopathologic grade	1	447	1.3	[0.6, 3.0]	3.3	[1.9, 5.9]	< 0.001
	2	3332	4.3	[3.6, 5.0]	6.6	[5.8, 7.6]	
	3/4	1497	8.8	[7.5, 10.3]	11.4	[9.9, 13.3]	
	missing	35	11.4	[4.5, 29.2]	15.5	[6.7, 36.0]	
HR expression	positive^a^	4132	4.1	[3.6, 4.8]	6.6	[5.8, 7.4]	< 0.001
	negative	775	12.4	[10.3, 15.0]	15.2	[12.8, 18.1]	
	missing	404	4.2	[2.7, 6.8]	5.2	[3.4, 8.0]	
HER2/neu expression	positive^b^	972	7.1	[5.7, 8.9]	10.8	[8.8, 13.2]	0.004
	negative	2941	4.9	[4.2, 5.7]	6.9	[6.0, 8.0]	
	missing	1398	5.1	[4.1, 6.4]	7.6	[6.3, 9.2]	
Subtype based on immunohistochemical surrogates	‘HR positive‘	4132	4.1	[3.6, 4.8]	6.6	[5.8, 7.4]	< 0.001
	‚HER2/neu positive‘^c^	234	12.9	[9.2, 18.0]	16.2	[11.8, 22.3]	
	‘triple negative‘^d^	376	12.0	[9.1, 15.8]	13.8	[10.6, 17.9]	
	missing	569	6.7	[4.9, 9.1]	9.1	[6.9, 11.9]	
BCS	T1/2N0	198	3.6	[1.7, 7.4]	4.9	[2.3, 10.2]	
BCS + RT	T1/2N0	2114	2.1	[1.6, 2.9]	4.4	[3.5, 5.5]	
Mastectomy	T1/2N0	606	4.8	[3.4, 6.9]	7.9	[5.9, 10.5]	
(BCS + RT or mastectomy) + HT	T1/2N0 HR positive ^a^	1749	1.7	[1.2, 2.4]	4.3	[3.3, 5.5]	
(BCS + RT or mastectomy) + CT	T1/2N0 HR negative	277	6.5	[4.2, 10.2]	9.3	[6.3, 13.7]	
(BCS + RT or mastectomy) + HT (if HR positive) + CT (if HR negative) + TT	T1/2N0 HER2/neu positive	73	0.0	–	0.0	–	
(BCS or mastectomy) + RT + CT + HT	T1/2N+ HR positive ^a^	554	5.8	[4.2, 8.2]	8.3	[6.2, 11.2]	
(BCS or mastectomy) + RT + CT	T1/2N+ HR negative	156	12.9	[8.5, 19.4]	16.5	[11.3, 24.1]	
(BCS or mastectomy) + RT + CT + HT (if HR positive) + TT	T1/2N+ HER2/neu positive	51	9.8	[4.2, 22,7]	9.8	[4.2, 22,7]	
MAST + RT + CT + HT	T3/4 HR positive ^a^	88	17.0	[10.7, 27.1]	17.0	[10.7, 27.1]	
MAST + RT + CT	T3/4 HR negative	50	22.0	[13.0, 37.3]	26.2	[16.3, 42.1]	

The sample includes female patients with primary invasive breast cancer (ICD-10 code: C50) without distant metastases from Saarland diagnosed between 1999 and 2009. *CI* cumulative incidence, *PE* point estimate of CI, *95% CI* 95% confidence interval of PE, *T, N* T and N category of TNM classification, *HR* hormone receptor, *HER2/neu* human epidermal growth factor receptor 2, *BCS* breast conserving surgery, *RT* radiotherapy, *CT* chemotherapy, *HT* hormonal (anti-estrogen) treatment, *TT* targeted treatment (trastuzumab). ^a^includes tumors with mixed HR expression (either estrogen or progesterone receptor positive), ^b^includes tumors with borderline expression of HER2/neu, ^c^HER2/neu positive, but HR negative, ^d^both HR negative and HER2/neu negative

**Table 6:** Five- and 10-year cumulative incidence of distant metastases of breast cancer patients with local R0 resection by age, tumor characteristics and provided recommended treatments

Category/treatment		*N*	5-year CI		10-year CI		*p*-value
			PE	95% CI	PE	95% CI	
Overall		5311	7.2	[6.5, 7.9]	11.3	[10.4, 12.3]	
Age	< 70 years	3714	7.8	[7.0, 8.7]	12.5	[11.4, 13.7]	< 0.001
	≥ 70 years	1597	5.8	[4.7, 7.0]	8.6	[7.2, 10.2]	
Clinical stage	T1/2N0	2918	3.6	[3.0, 4.4]	6.0	[5.1, 7.0]	< 0.001
	T1/2N+	1613	10.8	[9.4, 12.4]	17.5	[15.6, 19.7]	
	T3/4N0	124	11.3	[6.9, 18.6]	16.6	[10.9, 25.4]	
	T3/4N+	409	19.6	[16.1, 23.8]	27.3	[23.0, 32.4]	
	missing	247	3.3	[1.6, 6.5]	4.3	[2.3, 7.9]	
Morphology	invasive ductal	3690	7.6	[6.8, 8.5]	11.5	[10.4, 12.7]	0.085
	invasive lobular	758	6.6	[5.1, 8.7]	12.4	[10.1, 15.4]	
	mixed type	459	6.3	[4.5, 9.0]	11.3	[8.6, 15.0]	
	other	404	5.3	[3.5, 8.0]	7.0	[4.8, 10.2]	
Histopathologic grade	1	447	1.4	[0.6, 3.0]	3.9	[2.3, 6.6]	< 0.001
	2	3332	5.8	[5.0, 6.6]	9.8	[8.7, 10.9]	
	3/4	1497	12.1	[10.5, 13.8]	17.0	[15.1, 19.1]	
	missing	35	9.0	[3.0, 26.9]	9.0	[3.0, 26.9]	
HR expression	positive ^a^	4132	5.6	[5.0, 6.4]	10.0	[9.0, 11.0]	< 0.001
	negative	775	15.9	[13.5, 18.7]	19.0	[16.4, 22.1]	
	missing	404	6.3	[4.3, 9.1]	9.8	[7.0, 13.8]	
HER2/neu expression	positive ^b^	972	9.1	[7.4, 11.1]	15.1	[12.8, 17.8]	0.001
	negative	2941	6.6	[5.7, 7.5]	9.8	[8.7, 11.0]	
	missing	1398	7.2	[6.0, 8.7]	11.6	[9.9, 13.5]	
Subtype based on immunohistochemical surrogates	’HR positive‘	4132	5.6	[5.0, 6.4]	10.0	[9.0, 11.0]	< 0.001
	‚HER2/neu positive‘^c^	234	15.9	[11.8, 21.4]	19.4	[14.7, 25.5]	
	‚triple negative‘^d^	376	16.3	[12.9, 20.5]	18.7	[15.0, 23.2]	
	missing	569	8.9	[6.8, 11.5]	12.6	[10.0, 15.9]	
BCS	T1/2N0	198	3.1	[1.4, 6.7]	4.4	[2.2, 8.7]	
BCS + RT	T1/2N0	2114	3.0	[2.4, 3.9]	5.4	[4.5, 6.6]	
Mastectomy	T1/2N0	606	5.8	[4.2, 8.0]	8.2	[6.2, 10.8]	
(BCS + RT or mastectomy) + HT	T1/2N0 HR positive ^a^	1749	2.7	[2.0, 3.6]	5.2	[4.2, 6.5]	
(BCS + RT or mastectomy) + CT	T1/2N0 HR negative	277	7.9	[5.3, 11.9]	9.2	[6.3, 13.4]	
(BCS + RT or mastectomy) + HT (if HR positive) + CT (if HR negative) + TT	T1/2N0 HER2/neu positive	73	2.7	[0.7, 10.9]	2.7	[0.7, 10.9]	
(BCS or mastectomy) + RT + CT + HT	T1/2N+ HR positive ^a^	554	8.6	[6.5, 11.3]	15.4	[12.2, 19.3]	
(BCS or mastectomy) + RT + CT	T1/2N+ HR negative	156	23.2	[17.4, 31.0]	30.7	[23.9, 39.4]	
(BCS or mastectomy) + RT + CT + HT (if HR positive) + TT	T1/2N+ HER2/neu positive	51	13.7	[6.8, 27.5]	16.0	[8.4, 30.5]	
MAST + RT + CT + HT	T3/4 HR positive ^a^	88	14.8	[8.9, 24.5]	24.1	[15.8, 36.8]	
MAST + RT + CT	T3/4 HR negative	50	40.0	[28.3, 56.5]	40.0	[28.3, 56.5]	

The sample includes female patients with primary invasive breast cancer (ICD-10 code: C50) without distant metastases from Saarland diagnosed between 1999 and 2009. *CI* cumulative incidence, *PE* point estimate of CI, *95% CI* 95% confidence interval of PE, *T, N* T and N category of TNM classification, *HR* hormone receptor, *HER2/neu* human epidermal growth factor receptor 2, *BCS* breast conserving surgery, *RT* radiotherapy, *CT* chemotherapy, *HT* hormonal (anti-estrogen) treatment, *TT* targeted treatment (trastuzumab). ^a^includes tumors with mixed HR expression (either estrogen or progesterone receptor positive), ^b^includes tumors with borderline expression of HER2/neu, ^c^HER2/neu positive, but HR negative, ^d^both HR negative and HER2/neu negative

## Discussion

This study derived population-based long-term estimates of the risk of cancer recurrence of BRC patients from Germany who had received local surgery with free resection margins (R0) up to 10 years after diagnosis. Overall 5- and 10-year CI of BRC recurrence was 10 and 16%. The 10-year CI of loco-regional recurrence and distant metastases was 8 and 11%, respectively. The derived estimates showed substantial variation and were particularly increased if tumors were locally or regionally advanced, of high grade, or classified as subtype ‘HER2/neu positive’ (without HR expression) or ‘triple negative’.

To date, studies of the risk of loco-regional recurrence and distant metastases using population-based samples of BRC patients are scant. Published CPGs such as the European Society for Medical Oncology clinical practice guidelines [24], the American Society of Breast Surgeons consensus guideline [31], and the German interdisciplinary S3 guidelines [12] mostly refer to studies which used hospital-based cohorts [7, 13–15] or participants of clinical trials [4, 32]. Furthermore, several of these studies included patients who had been diagnosed decades ago (e. g. two studies from the US reported a 5-year risk of local recurrence of 8% of BRC patients who had been treated with lumpectomy and radiotherapy between 1968 and 1984 [13] or a 10-year risk of loco-regional recurrence and distant metastases of 17 and 35% of BRC patients who had been treated between 1975 and 1994 with mastectomy, chemotherapy and with or without hormonal therapy, respectively [4], and a more recently published study from the Netherlands reported a 5-year risk of BRC recurrence of 29 and 11% of patients with T1 tumors aged ≤40 and > 40 who had received BCS and radiotherapy between 1984 and 1997, respectively [15]). Therefore, the results of these studies may be outdated and not representative for more recently treated patients. Studies including participants of clinical trials (e.g. [4, 32]) may have even less external validity, as trial enrollees are often highly selected and therefore little representative of the overall population of BRC patients [33, 34].

To systematically identify registry-based studies presenting results of more recently diagnosed patients, the PubMed database was searched using the terms ‘breast cancer’, ‘recurrence’, and ‘registry’. Few studies from the Netherlands [35–39], the US [40], Canada [41], Italy [42] could be identified upon careful reading. However, several issues hampered the comparison of these studies’ findings with the results presented in this article. First, selective referral to participating clinics may have limited the applicability of the results derived from hospital-based cohorts (e.g. [7, 35, 40, 43–45]) to unselected populations of BRC patients. Second, some population-based studies investigated patient samples with special characteristics (e.g. patients with T1/2N0 basal like BRC subtype [36] or patients aged ≤35 years who received post-mastectomy radiation [41]), and corresponding subgroups of patients could not be analyzed in this study due to small patient numbers or missing data items. Third, some published population-based studies used ‘classical’ Kaplan-Meier estimators, reported proportions, or did not mention whether competing risks had been taken into account (e.g. [7, 37, 39, 40]).

A population-based study from the Netherlands reported similar overall 5-year risks of loco-regional recurrence and distant metastases of 4 and 9% of BRC patients who received surgery between 2003 and 2008 and who had a comparable stage distribution [37]. A recently published study from Denmark which linked data from different registries reported 5-year CI of BRC recurrence and bone and visceral metastases of 18, 2 and 5%, respectively, in a sample of 23,478 patients with regional or stage II or III BRC [46]. Patients of the current study with regional or stage II or III BRC who were overall comparable to the Danish sample had a 5-year CI of BRC recurrence and distant metastases of 16 and 11%, respectively (data not shown). In both studies the overall risk of BRC recurrence was comparable. The somewhat lower risk of distant metastases derived in the Danish study could have resulted from both a possible underestimation of metastases [46] and a somewhat higher proportion of patients with involved lymph nodes in the current study. Another recently published study from Italy used population-based samples of BRC patients and reported comparable overall proportions of patients with tumors with HR and HER2/neu expression and accordingly, similar proportions of luminal (subtypes A, B, and HER2/neu), HER2/neu like and basal like BRC of 83, 7, and 11%, respectively [42]. Observed 5-year risks of BRC recurrence of the Italian patients with luminal A, HER2/neu and basal like tumors of 7, 22, and 20% were quite comparable to this study’s results. A summary of the aforementioned and cited studies is provided as Additional file 1.

This study included virtually all women residing in the federal state of Saarland who had been diagnosed with primary invasive BRC between 1999 and 2009 [18, 19]. The derived estimates of BRC recurrence given in this work therefore reflect the risk of ‘real world’ patients and the effectiveness of cancer care provided in a routine setting. This study intended to investigate the risk of BRC recurrence of patients who had received surgery with free resection margins as a recommended local treatment [23, 25, 47]. The overall proportion of patients with involved resection margins after definitive surgery was 5% and these patients had a 2.4-fold increased risk of a metastatic spread of their tumor compared to patients with free resection margins.

Preceding analyses of the implementation of guideline recommendations in the study sample revealed that the proportion of patients (with available information on HER2/neu expression of their tumor) who received trastuzumab rose from 2 to 47% between 2000 and 2009 [48]. Thus, the observed findings of a 4.2-fold and a 1.5-fold increased risk of BRC recurrence of patients with early T1/2N0 and T1/2N+ BRC who had not received TT in addition to recommended local and other systemic treatments may be indicative for the effectiveness of trastuzumab therapy on a population level, even though the limited sample size and the observational design of this registry-based study must be taken into account.

One of the studies cited in the text and listed in Additional file 1 provided estimates of the effect of TT on the risk of BRC recurrence. Patients from The Netherlands with HER2/neu expressed tumors who had been treated with trastuzumab conveyed a 50% risk reduction of loco-regional BRC recurrence in the first 5 years after treatment compared to untreated patients [37]. The observed absolute risks of local and regional recurrence in that study were 2 and 1% among patients treated with trastuzumab and 4 and 3% among those without. In the current study, 5-year risk estimates of loco-regional recurrence of 6% among patients with and 7% among patients without trastuzumab treatment were observed in a patient sample which additionally encompassed patients with neoadjuvant systemic treatments.

The shapes of the curves of CI of BRC recurrence point to an initially higher hazard of patients with HR negative tumors which decreases over time and an initially lower but constant hazard of patients with HR positive tumors. Additional analyses confirmed these patterns and revealed a slightly higher hazard of BRC recurrence among patients with HR positive tumors starting 6 or 7 years after diagnosis (data not shown), which has been observed in participants of several clinical trials [23].

Recent studies have shown fear of recurrence to be highly prevalent among BRC survivors [49], and that many BRC patients overestimate their risk of cancer recurrence [50]. For these reasons, the presented results may extend available knowledge and have great relevance for clinicians, their patients and researchers. Furthermore, this study derived estimates of the long-term risk of BRC recurrence up to 10 years after diagnosis. To the best of the knowledge of the authors, so far only one study from the Netherlands had reported population-based long-term estimates of BRC recurrence of such an extended follow-up period [39]. This study has further strengths. First, the size of the study sample allowed to deriving detailed estimates by tumor characteristics and major treatment options. Second, the use of multiple sources of information ensured a high completeness and validity of the available data [48], i.e. detailed information of characteristics such as clinical stage, histopathologic grade, and subtype were available for 87, 97 and 85% of the tumors, respectively. The registration model with different sources of information and statutory provisions on the events to trigger cancer notifications, the interdisciplinary approach of the treatment of patients with recurrent BRC [22, 23] and the fact that recurrent and metastatic BRC often turns into a chronic condition maximized the likelihood of a registration of cancer recurrence and thus an almost complete follow-up of the patients with regard to these outcomes. Since the mid-1990s, the annual number of notifications of recurrent BRC to the Saarland CR was rather stable and ranged between 192 and 255, respectively.

However, there are also weaknesses that require careful consideration. Detailed data on administered local surgery and radiotherapy, chemotherapy, hormonal treatment and TT were available for 78, 82, 79, and 56% of the patients, respectively. Trastuzumab became available for treatment in the calendar period when the patients of this study received primary treatment. Therefore, the proportion of patients with information whether or not a TT was administered was lower compared to the other treatments. Despite this, the sample of patients used for the analyses showed great similarity with the study sample and was large enough to obtain estimates of the CI of BRC recurrence with sufficient precision for most of the clinically relevant subgroups of patients. As the majority of patients had received a guideline adherent treatment [48] and additional information, e.g. on co-morbidity of the patients was lacking, no comparative analyses of the risk of recurrence of patients with and without guideline adherent treatment have been carried out except for patients with early stage BRC showing HER2/neu expression. Furthermore, additional information on histopathological tumor characteristics which affect the risk of recurrence such as vessel invasion or prognostic marker Ki-67 [51] have not been collected or have come into routine use very recently only. The CR did not collect information of the staging examinations of individual patients. Major implications of different staging over time or an increasing sensitivity and improved accuracy of diagnostic procedures seem unlikely, as the observed proportion of patients with metastatic BRC at the time of diagnosis remained rather constant over time.

Based on recommendations of the National Cancer Plan from 2008, the majority of German federal states have meanwhile extended the existing population-based CRs to comprehensive CRs [52]. The collection of the data used in this study including cancer recurrence as outcome anticipated the items of the common and mandatory basic dataset which is now basis for data collection of comprehensive CRs in Germany. This common catalogue of data items, passive registration with defined events to trigger mandatory notifications to CRs and a sustainable funding hopefully prove to be suitable for collecting high quality CR data in Germany for reporting cancer burden, for monitoring the quality of cancer care and for oncologic research [52]. A recently published article which demonstrated the lack of reliable data on cancer recurrence after primary cancer treatment in the US and an accompanying editorial argued for the collection of such data by population-based CRs [53, 54]. The availability of population-based data of the long-term risk of cancer recurrence up to 10 years after diagnosis and derived from a much larger population of BRC patients from Germany is still some way off.

## Conclusions

The presented findings of this study may extend available knowledge of the long-term risk of cancer recurrence of an unselected population of BRC patients from a central European region who received cancer care in a routine setting. The study further demonstrated the usefulness of CRs for a population-based monitoring of the effectiveness of cancer care in terms of disease recurrence as a major treatment related outcome measure.

## Additional file

Additional file 1: Cited studies of the risk of cancer recurrence of breast cancer patients. (PDF 27 kb)

## Abbreviations

BCS: Breast conserving surgery
BRC: Breast cancer
CI: Cumulative incidence
CPG: Clinical practice guidelines
CR: Cancer registry
HER2/neu: Human epidermal growth factor receptor 2
HR: Hormone receptor
ICD-10: International Classification of Diseases, 10th Revision
RT: Radiation therapy
T, N, M: Categories of the UICC TNM staging classification
TT: Targeted treatment
