# Supplementary material for: Risk of loco-regional recurrence and distant metastases of patients with invasive breast cancer up to ten years after diagnosis – results from a registry-based study from Germany
Source: BMC Cancer. 2019 May 30;19:520. doi: 10.1186/s12885-019-5710-5 (PMC6543576; doi:10.1186/s12885-019-5710-5)
Supplement: Supplementary file 1 — Cited studies of the risk of cancer recurrence of breast cancer patients. (PDF 27 kb) [file 12885_2019_5710_MOESM1_ESM.pdf]

Risk of loco-regional recurrence and distant metastases of patients with invasive breast cancer up to ten years after diagnosis – results from a registry-based study from Germany

Additional file 1. Studies of the risk of cancer recurrence (loco-regional recurrence and distant metastases) of patients with invasive breast cancer (BRC) cited in clinical practice guidelines (category A), cited in the text presenting results derived from hospital-based samples or otherwise selected populations of patients (B) or presenting results derived from population-based samples (C) of BRC patients

Category	Origin of study sample	Country	Period of diagnosis	N	Age	Patient and tumor characteristics	Point estimates	Reference
A	clinical trial	US	1975-1994	1031	≤ 75 years	patients with stage II or IIIa tumors treated with mastectomy, chemotherapy and with or without hormonal therapy	10-year risk of loco-regional recurrence and distant metastases of 17% and 35%	[4]
A	hospital	US	1962-1984	433	All ages	patients with stage I and II tumors with BCS and RT	5-year risk of local recurrence of 8%	[13]
A	hospital	GB	1968-1976	603	All ages	patients with BCS or mastectomy with or without RT	3-year risk of cancer recurrence of 50%	[14]
A	hospital	NL	1984-1997	1085	All ages	patients with T1 tumors with BCS and RT	5-year risk of cancer recurrence of 29% (<= 40 years) and 11% (> 40 years)	[15]
A	clinical trial	US, GB, CA, SE, IT, DE, AT,	1976-1999	10,801	All ages	patients with or without RT after BCS	10-year risk of loco-regional recurrence of 35% (BCS) and 19% (BCS + RT)	[32]
B	hospital	KR	1999-2005	1153	All ages	patients without distant metastases receiving surgery with or without RT	5-year risk of cancer recurrence of 12%, 20%, 22% and 20% of patients with luminal A, luminal B, HER2/neu positive and triple negative tumors	[7]
B	hospital	NL	2003-2006	40,892	All ages	patients with T1-3M0 tumors	5-year risk of local recurrence ranged between 1% and 6%	[35]
B	health care program	US	2000-2006	234	30-90 years	patients with T1N0M0 and HER2/neu positive tumors	5-year risk of loco-regional and distant recurrence of 6%	[40]
B	hospital	DE	1988-2012	16,675	All ages	patients receiving mastectomy with or without subsequent RT	10-year risk of local recurrence of 9% (mastectomy with RT) and 12% (mastectomy alone)	[43]
B	hospital	US	1998-2011	1971	All ages	patients with triple negative tumors without distant metastases	5-year risk of local and regional recurrence of 4% and 2%	[45]

C	population-based CR	IT	2003-2005	3302	All ages	patients without distant metastases	5-year risk of loco-regional recurrence and distant metastases of 4% and 7%	[42]
C	population-based CR	NL	2003-2008	52,626	All ages	Patients without distant metastases and without neoadjuvant treatment	5-year risk of local recurrence, regional recurrence and distant metastases of 3%, 2%, and 9%	[37]
C	population-based CR	NL	2005-2008	2548	All ages	patients with triple negative T12N0 tumors without distant metastases with surgery	5-year risk of local recurrence, regional recurrence and distant metastases of 4%, 3% and 12%	[36]
C	population-based CR	NL	2003-2008	1000	20-35 years	patients with unilateral tumors without distant metastases and without neoadjuvant treatment who received surgery	5-year risk of local recurrence, regional recurrence and distant metastases of 4%, 4% and 14%	[38]
C	population-based CR	NL	2003	9342	All ages	patients with unilateral stage I-III tumors without involvement of skin or chest wall with local R0 resection without neoadjuvant systemic treatment	proportions of patients with local recurrence, regional recurrence and distant metastases of 4%, 2% and 14% after 10.25 yeas	[39]
C	population-based CR	CA	1994-2003	588	< 35 years	patients who received mastectomy	proportions of BRC recurrence of 48% after 8.6 years	[41]
C	linkage of different data population-based registries)	DK	1999-2012	23,478	all ages	patients with regional or stage II/III breast cancer who	5-year cumulative incidence of breast cancer recurrence,, visceral and bone metastases of 18%, 5% and 2%	[46]
C	population-based CR	DE	1999-2009	9359	All ages	patients without distant metastases who received surgery with local R0 resection	10-year risk of loco-regional, distant and overall BRC recurrence of 8%, 11% and 16%	current study

RT=radiotherapy, BCS=breast conserving surgery, CR=cancer registry, AT=Austria, CA=Canada, DE=Germany, DK=Denmark, GB=United Kingdom, IT=Italy, KR=Republic of Korea, NL=Netherlands, SE=Sweden, US=United States of America.
